# Maternity Pensions
**The Endowment of Motherhood.* By H. D. Harben. (London: The Fabian Society. 1910. Tract No. 149, pp. 23. Price 2d.)


**Published:** 1910-08-13

**Authors:** 


### The Hospital

A Journal of

The Medical Sciences and Hospital Administration.

New Series. No. 183, Vol. VII. [No. 1255, Vol. XLVIII.] SATURDAY, AUGUST 13, 1910

### Maternity Pensions.*

One need not necessarily be a Socialist to recognise that the conditions under which the average child of the people comes into this world are not ideal. Mr. Harben, who speaks with the enthusiasm which characterises the pronouncements of the Fabian brotherhood, will find public opinion in the main in agreement with him as regards the desirability of discovering a remedy for an admitted evil. Whether all will accept with equal agreement the remedy advocated is, however, quite a different matter. Maintaining that home life, at any rate in the old sense of the term, has already been more than half-destroyed by the modern industrial system, he points out that it is but a few steps in advance from the present condition of things—free education, free vaccination, free medical inspection, and free feeding of necessitous school children, to provide free State maintenance. He sees, however, two disadvantages to this ideal. The first is that the death-rate of infants in public institutions is distinctly higher than that in private homes, though statistics are always apt to be fallacious; and, again, the institutional solution savours somewhat of "regimentation" and might tend to produce a race cut to a standard pattern. The second great objection is that English opinion has gone almost as far as it will go in the direction of interference outside the home. A scheme which, while not replacing will reconstruct the home, is admittedly more likely to ensure the sympathy of public opinion. The first step in the proposed scheme must be the establishment of a system of complete public provision for all the extra expenses incident on maternity. The mother and child must be provided with adequate qualified medical and nursing attendance, followed by the provision of proper sustenance—fresh milk delivered daily in properly-sealed bottles. Free, universal, non-contributory maternity pensions should be provided. These should consist of ten shillings a week for eight weeks in ordinary cases, and should start two weeks before the expected date of the confinement, provided that the woman was *not* engaged at this time in any occupation likely to prove injurious to her health or her offspring. (It is difficult to understand the object of this last restriction. Surely it is precisely the woman engaged in an unhealthy occupation that stands most in need of being relieved of it by the provision of this preliminary pension?) The author arrives at the probable cost of his scheme by taking the 1907 return of births—namely, 1,148,573. A pension of ten shillings a week for eight weeks would therefore cost the community £4,600,000 per annum. Adding 10 per cent. extra for the cost of dealing with special cases would bring the total to £5,000,000. With the addition of £1,750,000 for the cost of provision of nursing, medical attendance, and milk, the total works out at £6,750,000. From this, however, would have to be deducted £1,350,000, the amount estimated to be saved by the fact that 20 per cent. of mothers would not claim the allowance under the scheme. The final cost would, therefore, be £5,400,000. In addition account must be taken of the indirect saving to the rates, charity, and the friendly societies.

It is impossible in the space at our disposal to criticise more than the purely medical side of the question. The author, under the cost of nursing and medical attendance, only allows 15s. per case as an average. This estimate he arrives at from figures supplied by the average cost of nursing and medical attendance to some unions and hospitals. But this calculation, we feel sure, is an under-estimate, unless the nursing and medical professions are once more to be sweated. Our institutions, both voluntary and rate-supported, are at present largely staffed by probationer nurses and newly-qualified doctors, but skilled assistance is always at hand when needed. In the case of home treatment, however, the assistance must be experienced and skilled from the first. It is quite obvious that rates of pay will have to be provided higher than those which are now accepted, on account of the experience to be acquired by probationers and newly-qualified medical men. The allowance of 15s. per case is, therefore, in our opinion quite inadequate. Moreover the rights of local practitioners will have to be safeguarded, for the appointment of a midwifery staff will certainly result in a diminution of their already hard-earned incomes, quite apart from the fact that they, in common with everyone else, will have to pay for the increased taxation which the adoption of such a scheme as Mr. Harben advocates will inevitably bring about.

Again, the estimated saving in charity is largely fanciful, for the supply of abnormal cases, such as constitute a large proportion of those admitted to lying-in hospitals, will not suddenly cease on the granting of a maternity pension. Also, under the scheme no provision is made for obtaining ante-partum advice such as is given to large numbers of out-patients at these hospitals. The need for these institutions will therefore remain the same as now, and no saving can be expected at least as regards these if the scheme were carried through. The author's sanguine estimate of the good effects on the infantile death-rate which this scheme would bring about are also largely imaginary. The fact that the death-rate in public institutions where the surroundings are the best obtainable is higher than that in the home should tend to show that heredity plays quite as important a part as environment in the production of a high death-rate. In fact, the balance of proof, as shown by Professor Karl Pearson,† lies rather on the side of heredity as the greater factor in this heavy mortality rate.

* *The Endowment of Motherhood.* By H. D. Harben. (London: The Fabian Society. 1910. Tract No. 149, pp. 23. Price 2d.)

† *Nature and Nurture.* By Karl Pearson, F.R.S. (London: Dulau and Co., Ltd. 1910. Price 1s. net.)

